# National trends in heart failure mortality in men and women, United Kingdom, 2000–2017

**DOI:** 10.1002/ejhf.1996

**Published:** 2020-09-23

**Authors:** Clare J. Taylor, José M. Ordóñez‐Mena, Nicholas R. Jones, Andrea K. Roalfe, Sarah Lay‐Flurrie, Tom Marshall, F.D. Richard Hobbs

**Affiliations:** ^1^ Nuffield Department of Primary Care Health Sciences University of Oxford Oxford UK; ^2^ NIHR Oxford Biomedical Research Centre Oxford UK; ^3^ Institute of Applied Health Research, University of Birmingham Birmingham UK

**Keywords:** Heart failure, Sex, Gender, Prognosis, Survival, Mortality

## Abstract

**Aims:**

To understand gender differences in the prognosis of women and men with heart failure, we compared mortality, cause of death and survival trends over time.

**Methods and results:**

We analysed UK primary care data for 26 725 women and 29 234 men over age 45 years with a new diagnosis of heart failure between 1 January 2000 and 31 December 2017 using the Clinical Practice Research Datalink, inpatient Hospital Episode Statistics and the Office for National Statistics death registry. Age‐specific overall survival and cause‐specific mortality rates were calculated by gender and year. During the study period 15 084 women and 15 822 men with heart failure died. Women were on average 5 years older at diagnosis (79.6 vs. 74.8 years). Median survival was lower in women compared to men (3.99 vs. 4.47 years), but women had a 14% age‐adjusted lower risk of all‐cause mortality [hazard ratio (HR) 0.86, 95% confidence interval (CI) 0.84–0.88]. Heart failure was equally likely to be cause of death in women and men (HR 1.03, 95% CI 0.96–1.12). There were modest improvements in survival for both genders, but these were greater in men. The reduction in mortality risk in women was greatest for those diagnosed in the community (HR 0.83, 95% CI 0.80–0.85).

**Conclusions:**

Women are diagnosed with heart failure older than men but have a better age‐adjusted prognosis. Survival gains were less in women over the last two decades. Addressing gender differences in heart failure diagnostic and treatment pathways should be a clinical and research priority.

## Introduction

Heart failure (HF) is a common syndrome in both women and men with high associated healthcare costs.^1^, ^2^ Incidence is strongly age‐dependent, and as life expectancy continues to improve, the prevalence of HF is likely to increase in both genders.^3^ However, gender‐specific differences in the epidemiology, pathophysiology and clinical presentation of HF have previously been reported.^4^ The incidence of HF is lower in women than men in younger age groups, but this trend reverses over the age of 80 years.^5^

The most common risk factor for HF in women is hypertension and in men is coronary artery disease.^6^ Women are more likely to develop HF with preserved ejection fraction (HFpEF), and men are more likely to develop HF with reduced ejection fraction (HFrEF).^7^ On echocardiography there are further anatomical and physiological differences such as lower left ventricular mass, stroke volume and ejection fraction in women, even once adjusted for age and body size.^8^ Guidelines recommend drug and device therapies regardless of gender but there is evidence that policy may not translate into practice.^9^, ^10^ Women are less likely to be investigated according to guideline‐specific pathways and receive target doses of evidence‐based drug treatments less often than men.^11^ The effect of these differences on overall survival in men and women is not fully understood. Prognosis following a diagnosis of HF is poor in both genders.^12^ Gender‐specific mortality rates have been reported in screened cohorts, hospital inpatients and trial subgroups^13^, ^14^ but gender‐specific long‐term outcomes among unfiltered community cohorts are relatively unknown. A recent review of sex differences in HF highlighted the pressing need for further research to better understand differences in prognosis, including how this relates to pathophysiology and treatment.^15^

In this study we aimed to use data from the SurviveHF population‐based cohort to report short‐ and long‐term mortality rates in a contemporary sample of women and men and explore cause of death and trends in survival over time.

## Methods

### Design and setting

The methods for the SurviveHF study have been described previously.^16^ The study protocol was approved by the Independent Scientific Advisory Committee to the Medicine and Healthcare products Regulatory Authority (protocol number 18_061R). An open retrospective population‐based cohort study was carried out using data from the Clinical Practice Research Datalink (CPRD) between 1 January 2000 and 31 December 2017. CPRD is a primary care database containing electronic patient records from over 700 general practices and is representative of the UK population. Practices that contributed at least one year of clinical data were included. Data quality measures included using the CPRD ‘acceptable’ patient flag, which is a simple quality check to ensure that the records used for research projects are as accurate and reliable as possible.

### Database linkage

CPRD data were linked to the Office for National Statistics (ONS) civil death registry to provide the date and cause of death according to the death certificate as completed by the examining physician. This is mandatory for all deaths in England and Wales. CPRD data were also linked to inpatient Hospital Episode Statistics (HES) to identify people admitted to hospital within 3 months of HF diagnosis (inpatient clinical code of HF and/or inpatient echocardiography report). Data linkage uses a deterministic matching algorithm, using the patient NHS number and at least one other unique identifier. This successfully matches more than 98% of ONS mortality data and 97% of HES records.

### Study population

The cohort included people with a diagnostic code of HF in their primary care record, aged 45 years and over, registered at an up‐to‐standard practice for at least 12 months and eligible for HES and ONS linkage. The NHS Clinical Terminology Browser, Quality and Outcomes Framework guideline and International Classification of Diseases (ICD) 10th Revision were used to generate a comprehensive list of codes for a diagnosis of HF. Previous studies have attempted to validate coding of HF in CPRD and suggest the population identified is similar to those in HF registries.^17^

Patients entered the cohort on the latest of the following dates: 1 January 2000, date of 45th birthday, patient registration date plus 12 months, practice up to standard date plus 12 months. Patients with a diagnosis of HF occurring before this date were excluded. Patients exited the cohort on the earliest of the following dates: 31 December 2017, patient transferred out date, date of death, last date of practice data collection, last date of available linked data.

Demographic variables including age, gender, ethnicity, patient level deprivation quintile as per the Index of Multiple Deprivation (IMD), cardiovascular risk factors and comorbidities were extracted for each participant. We refer to gender rather than sex as CPRD records how people self‐identify rather than biological sex, though in practice we suspect there would be only relatively small numbers of people who identify as a different gender to their biological sex and this would be unlikely to influence results given the large sample size. Cardiovascular risk factors [smoking, blood pressure, cholesterol, body mass index (BMI)] were the most recent recorded prior to the index date. Cardiovascular disease (CVD) comorbidities (angina, myocardial infarction, ischaemic heart disease, diabetes, hypertension, stroke, atrial fibrillation, valve disease) were defined by the presence of a clinical code at any time prior to the entry date.

### Outcomes

The primary outcome measure was death (all‐cause and cause‐specific mortality) in men and women. Secondary outcomes included primary cause of death due to HF (at any position in the cause of death hierarchy). Causes of death subgroups were defined using the ICD 9th and 10th Revision (online supplementary *Table* *S1*).

### Statistical analysis

The numbers of HF cases in men and women were calculated, and baseline characteristics presented for each group using means and standard deviations (SD) for continuous variables, and proportions and percentages for categorical variables. Causes of death occurring in less than 10% of men and women were grouped (with the exception of HF).

All‐cause, cardiovascular, and non‐cardiovascular mortality rates at 1, 5, 10 and 15 years were estimated for men and women with HF, and by each 10‐year age band over the age of 45, using cumulative incidence estimation in the presence of competing risk events.^18^

To investigate trends in overall survival over time, survival rates at 1, 5 and 10 years by year of diagnosis were estimated using the Kaplan–Meier method. Linear trends in the survival rates over time were investigated by fitting a weighted linear regression of the survival rate on the year of diagnosis in which the weights were inversely proportional to the variance of the survival rate. The difference in survival rates and cause‐specific mortality rates between the earliest and most recent years of diagnosis were computed and 95% confidence intervals (CI) were calculated using the normal distribution. The interaction between year of diagnosis and gender was tested for in a Cox proportional hazards regression.

Kaplan–Meier curves and log‐rank tests were used to compare crude survival in men and women with HF. Cox regression was used to estimate hazard ratios (HR) for the effect of gender on all‐cause and cause‐specific mortality, adjusting for potential confounders. Mixed modelling, adjusting for age and clustering of patients within practices, was used to confirm any observed association between gender and mortality over time. Further adjustment allowed for IMD quintile, ethnicity, cardiovascular risk factors and CVD comorbidities. The proportional hazards assumption was tested by plotting Schoenfeld residuals over time. No clear trends over time were evident for any of the covariates in the model. We coded a binary variable for hospitalisation (yes vs. no as reference). Hospitalisation at the time of diagnosis has been linked to a worse prognosis for people with HF and so we hypothesised that there may also be gender differences in all‐cause mortality based on hospitalisation at diagnosis.^16^ Kaplan–Meier was used to compare age‐specific survival between men and women in hospitalised and non‐hospitalised HF patients. Cox proportional hazards was used to test for effect modification by hospitalisation at diagnosis, by adding a multiplicative interaction term between gender and hospitalisation. We did not analyse differences in number of hospitalisations during follow‐up.

There was substantial missing data for cholesterol and BMI and comparison of the characteristics of those with and without missing data suggested the data were not missing at random.^19^, ^20^ Multiple imputation was therefore considered inappropriate and an alternative approach undertaken where continuous variables (BMI, cholesterol, systolic and diastolic blood pressure) were categorised and unrecorded data represented by an additional missing category. Complete case analysis was undertaken as sensitivity analyses, with and without cholesterol and BMI as covariates.

Statistical analysis was carried out using R (version 3.6.0) using ‘survival’, ‘survminer’, and ‘cmprsk’ packages.^21^, ^22^, ^23^, ^24^

## Results

In total, 55 959 incident HF cases were identified; 29 234 (52.2%) men and 26 725 (47.8%) women. Of these, 24 125 people (43.1%) were hospitalised around the time of diagnosis; 12 438 (42.5%) men and 11 687 (43.7%) women. The percentage of men and women requiring hospitalisation around the time of diagnosis increased between 2000 and 2007, then remained stable from 2008 onwards.

The baseline characteristics of men and women with HF are shown in *Table* *1* and online supplementary  Table *S2*. Comorbid CVD was common, with hypertension more prevalent among women and men more likely to have ischaemic heart disease, previous myocardial infarction, diabetes, or be smokers. The average age at diagnosis overall was 77.1 (SD 10.6) years and did not change over the study period. Women were on average almost 5 years older at diagnosis than men (79.6 years vs. 74.8 years). Amongst women with HF, 8.1% were diagnosed before the age of 65 and 34.4% diagnosed at age 85 years or older, compared to 17.8% of men diagnosed before 65 and 18.4% at age 85 years or older (*Table* *1*).

**Table 1:** Sociodemographic and clinical characteristics of men and women with heart failure at the time of diagnosis

Characteristic	Men	Women	*P*‐value
Overall, *n* (%)	29 234 (100)	26 725 (100)	
Place of diagnosis, *n* (%)			0.005
Primary care	16 796 (57.5)	15 038 (56.3)	
Hospital	12 438 (42.5)	11 687 (43.7)	
Age, years, mean ± SD	74.8 ± 10.6	79.6 ± 9.87	<0.001
Age category, *n* (%)			<0.001
45–64 years	5205 (17.8)	2159 (8.10)	
65–74 years	7671 (26.2)	4814 (18.0)	
75–84 years	10 977 (37.5)	10 557 (39.5)	
≥85 years	5381 (18.4)	9195 (34.4)	
Ethnic group, *n* (%)			<0.001
White	23 017 (78.7)	21 126 (79.0)	
Non‐white	845 (2.89)	652 (2.44)	
Mixed	3636 (12.4)	2949 (11.0)	
Index of deprivation, *n* (%)			<0.001
1 (least deprived)	5885 (20.1)	4969 (18.6)	
2	6876 (23.5)	6078 (22.7)	
3	6196 (21.2)	5751 (21.5)	
4	5972 (20.4)	5735 (21.5)	
5 (most deprived)	4282 (14.6)	4165 (15.6)	
Smoking status, *n* (%)			<0.001
Never	8018 (27.4)	13 234 (49.5)	
Former	4331 (14.8)	2763 (10.3)	
Current	15 661 (53.6)	8846 (33.1)	
SBP, mmHg, mean ± SD	135.4 ± 20.1	139.9 ± 21.7	<0.001
DBP, mmHg, mean ± SD	76.5 ± 11.6	77.3 ± 11.6	<0.001
Total cholesterol, mmol/L, mean ± SD	4.46 ± 4.7	4.98 ± 1.23	<0.001
BMI, kg/m^2^, mean ± SD	28.0 ± 5.39	27.8 ± 6.75	0.009
History, *n* (%)			
AF	7877 (26.9)	6752 (25.3)	<0.001
Angina	7133 (24.4)	4832 (18.1)	<0.001
Diabetes	7416 (25.4)	5688 (21.3)	<0.001
Hypertension	15 894 (54.4)	16 422 (61.4)	<0.001
IHD	9165 (31.4)	5441 (20.4)	<0.001
MI	7675 (26.3)	3621 (13.5)	<0.001
Other CVD	7571 (25.9)	6186 (23.1)	<0.001
Stroke	3441 (11.8)	2830 (10.6)	<0.001
Valve disease	2086 (7.14)	2068 (7.74)	0.007

AF, atrial fibrillation; BMI, body mass index; CVD, cardiovascular disease; DBP, diastolic blood pressure; IHD, ischaemic heart disease; MI, myocardial infarction; SBP, systolic blood pressure; SD, standard deviation.

Categories with missing data are reported in online supplementary *Table* *S2*.

### All‐cause mortality rates

There were 30 906 deaths during the study period: 15 822 in men and 15 084 in women. Crude mortality rates for all‐cause, cardiovascular and non‐cardiovascular mortality were higher in women than in men (online supplementary *Table* *S3*). However, age‐specific all‐cause mortality rates tended to be lower in women than men, though this was only statistically significant in the 75–84‐year‐old age group (*Table* *2*).

**Table 2:** Age specific all‐cause mortality rates^a^ at 1, 5, 10, and 15 years after a diagnosis of heart failure by gender and age category (expressed as a percentage of those who died)

Age category	1‐year MR	5‐year MR	10‐year MR	15‐year MR
45–64 years				
Men	11.5 (10.7–12.4)	22.6 (21.5–23.7)	29.2 (28.0–30.5)	30.9 (29.7–32.2)
Women	10.3 (9.04–11.6)	23.1 (21.3–24.8)	29.8 (27.9–31.8)	32.1 (30.2–34.1)
65–74 years				
Men	16.2 (15.3–17.0)	35.2 (34.1–36.3)	45.9 (44.8–47.0)	48.7 (47.6–49.8)
Women	16.1 (15.0–17.1)	33.6 (32.3–34.9)	43.5 (42.1–44.9)	46.2 (44.8–47.6)
75–84 years				
Men	24.2 (23.4–25.0)	50.4 (49.4–51.3)	59.9 (58.9–60.8)	61.3 (60.4–62.2)
Women	21.2 (20.4–22.0)	44.0 (43.0–44.9)	54.2 (53.3–55.2)	56.0 (55.1–57.0)
≥85 years				
Men	36.4 (35.2–37.7)	64.8 (63.5–66.0)	–	–
Women	36.3 (35.4–37.3)	61.9 (61.0–62.9)	–	–

MR, mortality rate.

a MR refers to the percentage of the baseline population who have died at each time point.

The median crude survival time for men with HF was 4.47 years (95% CI 4.35–4.58) compared to 3.99 years (95% CI 3.89–4.10) for women. Cox regression unadjusted analysis suggested that overall women with HF had a 10% higher risk of death from all causes than men (HR 1.10, 95% CI 1.08–1.13). After adjustment for age, women with HF were at a 14% lower risk of death from all causes (HR 0.86, 95% CI 0.84–0.88). Similar results were observed in the results of the model adjusted for all additional potential confounders using a complete case analysis (HR 0.90, 95% CI 0.87–0.93) and categorising continuous variables and adding missing categories (HR 0.87, 95% CI 0.84–0.89).

### Cause of death

The causes of death for men and women are shown in *Table* *3*. For men and women combined, HF was the primary cause of death in 2237 (7.2%) but listed as any cause in 13 093 (42.4%). In unadjusted analyses, women were more likely to die from HF than men (HR 1.53, 95% CI 1.42–1.65), but after adjustment for age this effect disappeared (HR 1.03, 95% CI 0.96–1.12). Similarly, in age‐adjusted Cox regression analyses, women were less likely to die from both CVD (HR 0.85, 95% CI 0.82–0.87) and non‐CVD (HR 0.87, 95% CI 0.84–0.90) compared to men, though the opposite was true in unadjusted analyses. Age and gender specific CVD and non‐CVD mortality rates are shown in online supplementary *Table* *S4*.

**Table 3:** Cause of death in men and women with heart failure

Cause of death subgroup	Men, *n* (%)	Women, *n* (%)
Diseases of the circulatory system	8883 (56.1)	8324 (55.2)
Heart failure, primary cause	965 (6.10)	1272 (8.43)
Heart failure, any cause of death^a^	6618 (41.8)	6475 (42.9)
Diseases of the respiratory system	2461 (15.6)	2464 (16.3)
Neoplasms	2295 (14.5)	1559 (10.3)
Other causes of death	2183 (13.8)	2737 (18.1)

a Includes patients for whom heart failure may have been the primary, or a contributory cause of death. Subgroups accounting for more than 10% of all deaths are reported.

### Trends in overall survival over time

**Figure 1:**
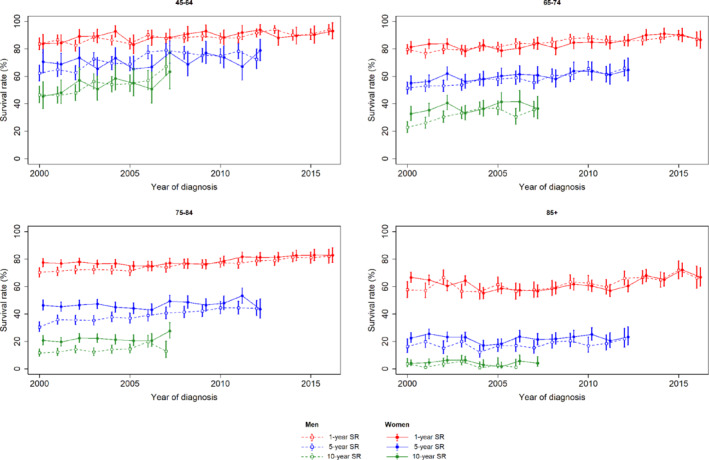
Comparison of overall survival rates (SR) at 1, 5, and 10 years after diagnoses of heart failure for men and women, according to the year of diagnosis and stratified by age category.

Survival rates were better for women at the start of the study period. In both men and women, overall survival improved over time (online supplementary *Figure* *S1*). In men with a new diagnosis of HF, the crude 1‐, 5‐ and 10‐year survival rates improved by 8.9% (95% CI 5.3–10.8), 11.1% (95% CI 7.1–13.2) and 7.7% (95% CI 4.0–9.6), respectively, across the study period. For women, the comparative improvements were 4.1% (95% CI 0.2–6.1), 2.3% (95% CI −2.3 to 4.6) and 4.7% (95% CI 0.7–6.8). Age‐specific overall survival rates improved over time for both men and women, particularly in younger age groups (*Figure* *1*). There was a trend towards greater improvements in survival for men across all age groups and duration of follow‐up (online supplementary *Table* *S5*). The interaction between year of diagnosis and gender was significant (*P* < 0.001). Between 2000 and 2016, per year of diagnosis all‐cause mortality was reduced by 2.9% in men (HR 0.971, 95% CI 0.967–0.975) and 1.4% in women (HR 0.986, 95% CI 0.982–0.990).

### Trends in cause‐specific mortality over time

**Figure 2:**
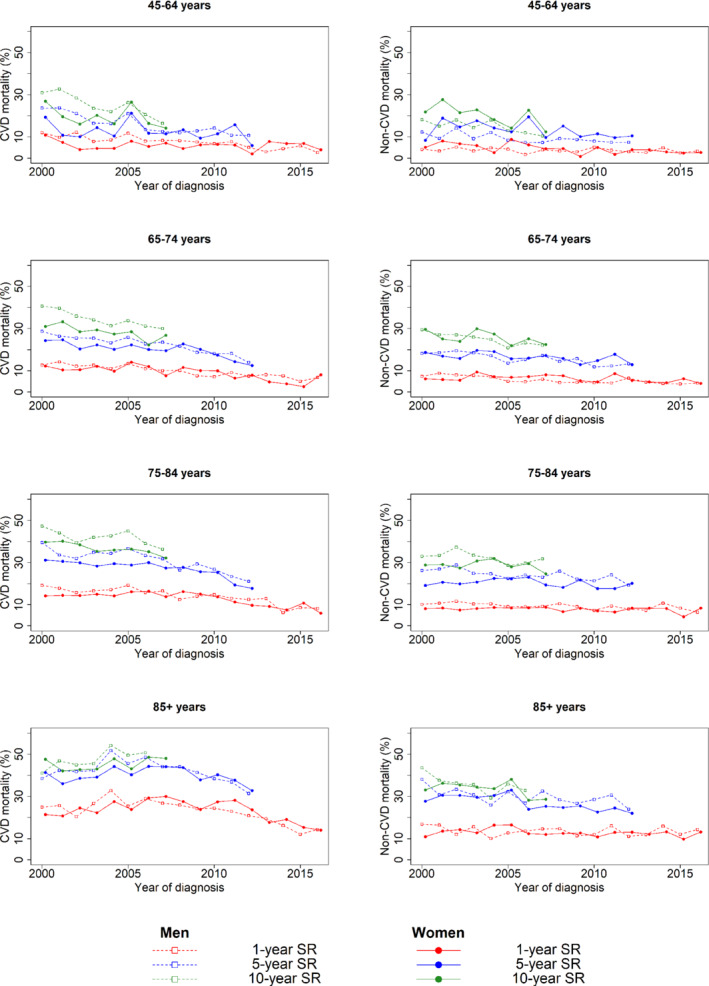
Comparison of overall survival rate (SR) between men and women since baseline (heart failure diagnosis) stratified by age category at diagnosis (in each row), and cause of death (cardiovascular vs. non‐cardiovascular in the left and right column, respectively). CVD, cardiovascular disease.

Over time, there was a decline in both age‐specific (*Figure* *2*) and crude CVD mortality rates (online supplementary *Figure* *S2*) at 1, 5 and 10 years. There were no clear differences between men and women. In men with a new diagnosis of HF, 1‐, 5‐ and 10‐year CVD mortality rates decreased across the study period by 8.8% (95% CI 6.2–10.1), 14% (95% CI 11.1–15.5) and 8.7% (95% CI 5.5–10.4) compared to respective changes of 7% (95% CI 4.4–8.4), 11.4% (95% CI 8.4–12.9) and 4.5% (95% CI 1.2–6.2) in women.

### Overall survival in men and women by hospitalisation at the time of diagnosis

**Figure 3:**
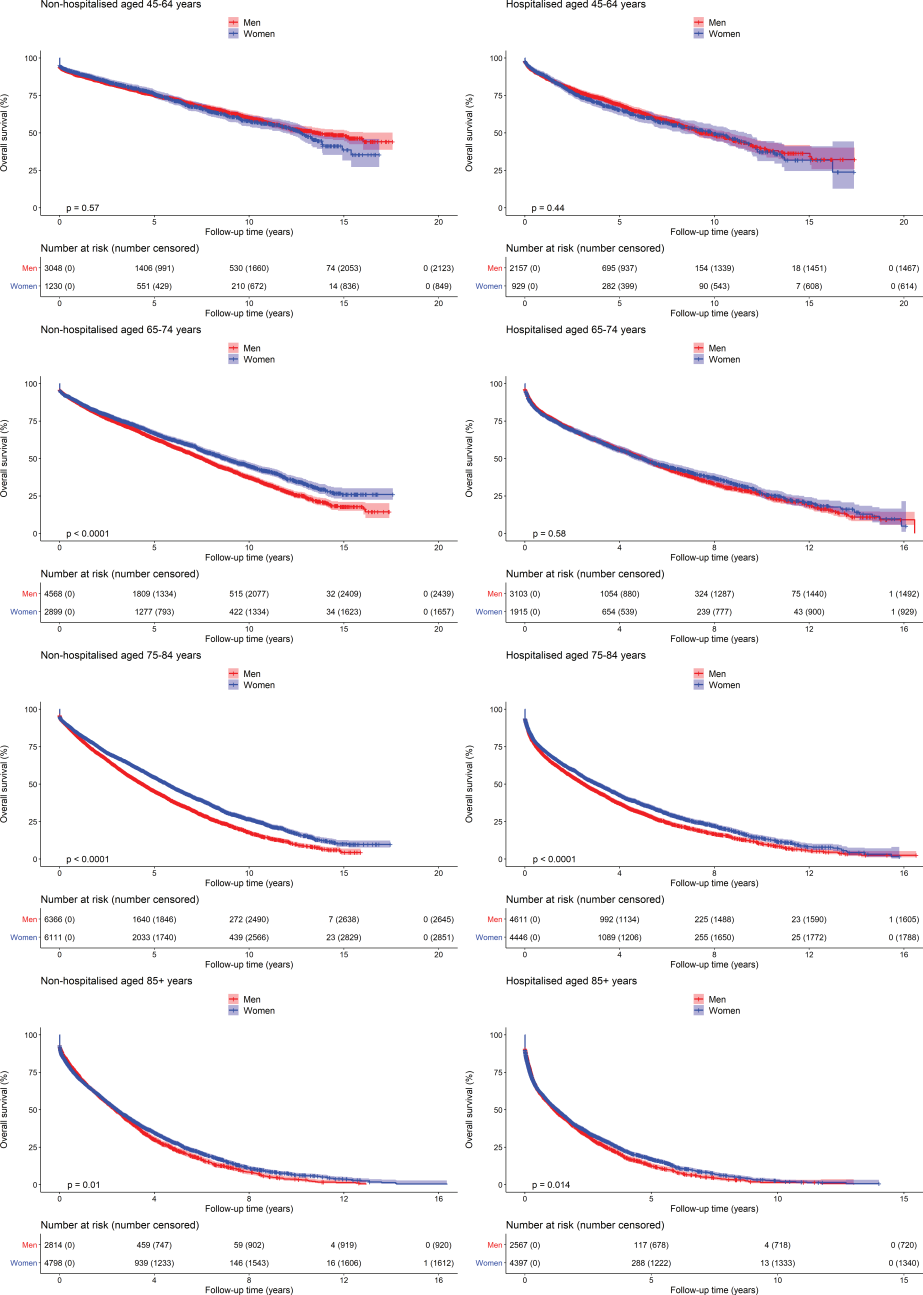
Comparison of overall survival rate between men and women since heart failure diagnosis stratified by age at diagnosis (in each row), and place of diagnosis (non‐hospitalised vs. hospitalised in the left and right column, respectively).

The difference in overall survival between men and women was more marked in patients diagnosed in clinic rather than at time of an acute hospital admission (online supplementary *Table* *S6*). This was particularly visible in the people aged between 65–74 and 75–84 years compared to those <65 or ≥85 years (*Figure* *3*). A significant interaction between hospitalisation and gender was observed (*P* = 0.02). This means that the reduction in age‐adjusted all‐cause mortality risk among women compared to men, was slightly greater among those diagnosed in the community (HR 0.83, 95% CI 0.80–0.85) than among those hospitalised (HR 0.89, 95% CI 0.86–0.92).

## Discussion

This large population‐based cohort, representative of the general population in the UK and with data from the millennium to the end of 2017, allowed short‐ and long‐term overall survival and cause‐specific mortality rate estimates to be compared for men and women with HF. Overall, when taking into account age at diagnosis, women had a better prognosis than men and were less likely to die from CVD but not HF. Both men and women with HF saw modest but significant improvements in overall survival at 1‐, 5‐ and 10‐year follow‐up. However, across the study period survival gains were greater in men than women. There was also a significant interaction between gender and hospitalisation, with survival greater in women than men when HF was diagnosed in the community but not when it was diagnosed at the time of a hospital admission.

A recent study reported sex differences in 1‐year survival among 90 707 people with HF in an ambulatory setting. In 2013, women had an age‐standardised mortality of 85 per 1000 vs. 83 per 1000 in men but once adjusted for other risk factors, female sex was associated with a mild reduction in risk of all‐cause mortality (HR 0.97, 95% CI 0.93–1.00).^25^ Our results demonstrate a similar pattern over a much longer follow‐up period, with unadjusted mortality higher among women with HF, but men at higher risk of death in age‐adjusted analyses. A survival analysis using North American registry data looked at trends in HF survival and hospitalisation between 1993 and 2014.^26^ There was a significant reduction in HF hospitalisation and an age‐adjusted reduction in HF mortality in most states, but no improvement in population‐level or gender‐specific HF‐related mortality across the study period.^26^ Differences in healthcare delivery and changing demographics of people with HF over time may in part explain the differences in results from our cohort, where survival for people with HF has improved over a similar time period. In keeping with findings from previous studies, average age at time of diagnosis did not change for either men or women in our study.^3^, ^16^

The limited improvement in survival rates for women compared to men in our study is concerning. Prevalence of HFrEF is higher in men and improvement in survival in men may be due to benefits from disease‐modifying medication.^7^ The prescribing of these medications has been increasing over the study time period and may account for some of the additional improvement in survival seen in men. For women, HFpEF is more prevalent,^27^ with recent reports suggesting a growing gap in the incidence of HFrEF in men and HFpEF in women over time.^15^ The absence of disease‐modifying agents for the treatment of HFpEF may therefore explain the limited improvement in survival among women.

Differences in survival may also reflect previously identified differences between genders in accessing diagnostic and treatment pathways.^11^ A recent study exploring first diagnosis of HF, also using CPRD, found that women waited longer than men for referral to specialist services (262 vs. 210 days, *P* = 0.001), diagnosis (1052 vs. 882 days, *P* < 0.001) and medical treatment (889 vs. 710 days, *P* < 0.001).^28^ Women with HFrEF are less likely to receive mineralocorticoid receptor antagonists or reach optimal doses of HF medical therapy when compared to men.^29^ Future individual patient level research could help to determine the reason behind these apparent differences, but it is possible that delays in diagnosis and treatment may have adversely impacted survival gains in our study population.

International HF guidelines currently recommend broadly the same approach to diagnosis and treatment for men and women. However, emerging evidence suggests future HF management may need to be tailored by gender. Differences in pharmacokinetics and pharmacodynamics mean the same dose of HF medication can result in twice the plasma drug concentration in women compared to men.^30^ A recent post‐hoc analysis of the BIOSTAT‐CHF study found for women the primary outcome of time to all‐cause mortality or HF‐related hospitalisation was lowest at 50% of the guideline‐recommended angiotensin‐converting enzyme (ACE) inhibitor and beta‐blocker dose, with no further decrease in risk at higher dosages.^31^ Women with asymptomatic left ventricular systolic dysfunction may not benefit from ACE inhibitors, though men do.^32^ Whilst we have not analysed treatment rates, the findings of these studies may in part explain why reductions in mortality have been greater in men than women since the widespread use of medications such as ACE inhibitors and beta‐blockers.

### Strengths and limitations

The use of routinely collected data allows a unique insight into outcomes within real‐life healthcare settings. Healthcare in the UK is provided to the entire population through registration with a primary care provider, meaning the study participants are broadly representative of the wider public. This study used primary and secondary care data sources to reliably estimate survival following diagnosis.

Mortality data, linked via the ONS, was taken directly from death certificate details entered by the treating physician which is used in national statistics in the UK and is the most reliable data source available.^33^ However, death certificate diagnoses are prone to inaccuracies, with around one third or more differing to the reported cause of death from autopsy findings.^34^ CVD seems to be over‐reported on death certificates, often by as much as 20%, and this might be reflected in the cause of mortality data we report.^34^ Physicians primarily record data for the purpose of medical care and this can lead to incomplete data, though CRPD has been shown to be a reliable source for diagnostic codes.^35^ We also used secondary care data to confirm HF diagnostic codes where available. Coding did not allow us to analyse HF classification based on left ventricular ejection fraction, underlying cause of HF or New York Heart Association class. For example, only 5.8% (*n* = 3342) of all participants were coded as either HFrEF or HFpEF. This is an important limitation when interpreting the different trends in survival and understanding the potential impact of treatment on reported outcomes. People with HFrEF have nearly double the risk of death over long‐term follow‐up compared to those with HFpEF in age and sex‐adjusted analyses.^36^

We did not extract data on treatments, device therapy, or transplantation as our aim was to report overall survival rates in men and women over a long period of time and interpreting these data without left ventricular ejection fraction classification would be flawed. We did not collect data on biomarkers such as N‐terminal pro B‐type natriuretic peptide or creatinine, though we recognise these can be important prognostic markers. Lead‐time bias could explain some of the trend to improving survival over time, though we suspect this should be equal between genders.

We report our results in relation to gender rather than sex because UK primary care records are based on self‐identified and reported gender. It was not possible to disaggregate survival mediators by sex and gender using these observational data, capture non‐binary gender, nor to assess for a sex‐gender interaction effect. We have reported our findings with reference to the Sex and Gender Equality in Research (SAGER) guidelines and sought to be transparent about the use and rationale for the terminology used but believe this is an area that merits further research.^37^

Multimorbidity is common among people with HF, with previous research demonstrating that the majority of people with HF have three or more other long‐term health problems.^3^ Survival time following a diagnosis of HF will be dependent on a range of individual patient factors, some of which will not be directly related to HF. Analyses of population level data cannot account for factors such as shared decision making and individual patient choice that will have influenced treatment, though there is no reason to suspect this would account for the difference in survival seen between genders. Whilst our results highlight important differences in outcomes and trends in survival between men and women, person‐centred care remains central to providing the best treatment for individuals, recognising the importance of quality of life as well as overall survival time.

### Implications

The findings of this study have important implications for future research and healthcare policy. There is a need for greater understanding of the physiological differences between men and women with HF. The dramatic improvements in recovery seen among women with certain types of HF, such as idiopathic dilated cardiomyopathy, offers hope that further research in this area may lead to future treatments that recover myocardial function.^38^ Diagnostic pathways should be further explored to ensure equality of access to imaging, specialist assessment and all treatment modalities. Improving future care for both men and women will require optimisation of treatment, particularly those of proven prognostic benefit in HFrEF.

## Conclusions

Women develop HF 5 years later than men but have a better age‐adjusted prognosis following diagnosis. Modest improvements in survival since the millennium have been seen in men but less so in women with HF. Our results highlight the need to research and address gender differences in risk factor management, diagnosis and implementation of prognostically beneficial treatments to improve HF survival for both men and women.

### Funding

The study was funded by the National Institute for Health Research (NIHR) Collaboration for Leadership in Applied Health Research and Care (CLAHRC) Oxford at Oxford Health NHS Foundation Trust and the Wellcome Institutional Strategic Fund. The funders did not have any role in the design of the study, analysis and interpretation of the data, or writing of the results for publication. C.T. is a NIHR Academic Clinical Lecturer. A.R., S.L.F. and J.M.O.M. are supported by the NIHR Biomedical Research Centre Oxford (BRC) Oxford University Hospitals NHS Foundation Trust. J.M.O.M. is also supported by the NIHR Community Healthcare Medtech and In Vitro Diagnostics Cooperative (MIC). N.J. is a Wellcome Trust Doctoral Research Fellow (grant number 203921/Z/16/Z). T.M. is funded by the CLAHRC West Midlands. F.D.R.H. acknowledges support from the NIHR School for Primary Care Research, NIHR CLAHRC Oxford, the NIHR Oxford BRC and Harris Manchester College, Oxford. The views expressed are those of the authors and not necessarily those of the NHS, the NIHR, or the Department of Health and Social Care.

This work uses data provided by patients and collected by the NHS as part of their care and support and would not have been possible without access to this data. The NIHR recognises and values the role of patient data, securely accessed and stored, both in underpinning and leading to improvements in research and care.

**Conflict of interest:** C.T. reports speaker fees from Vifor and Novartis and non‐financial support from Roche outside the submitted work. J.M.O.M., A.R. and S.L.F. report grants from the NIHR BRC Oxford. N.J. reports a grant from the Wellcome Trust. T.M. reports a grant from CLAHRC West Midlands. F.D.R.H. reports personal fees and other from Novartis and Boehringer Ingelheim, and grants from Pfizer outside the submitted work.

## Supporting information


**Table S1.** International Classification of Diseases, 9th and 10th revision codes.
**Table S2.** Missing data for sociodemographic and clinical characteristics of men and women with heart failure at the time of diagnosis.
**Table S3.** Mortality rates at 1, 5, 10, and 15 years after a diagnosis of heart failure by gender.
**Table S4.** Cardiovascular disease (CVD) and non‐CVD mortality rates at 1, 5, 10, and 15 years after a diagnosis of heart failure by gender and age category.
**Table S5.** Improvements in the survival rate over time for men and women across age groups.
**Table S6.** Crude survival rates at 1, 5, 10, and 15 years after a diagnosis of heart failure by gender and hospitalisation at time of diagnosis.
**Figure S1.** One, 5 and 10‐year survival for men and women with heart failure by year of diagnosis.
**Figure S2.** Cause‐specific mortality rates over time by gender.
