# Supplementary material for: National trends in heart failure mortality in men and women, United Kingdom, 2000–2017
Source: Eur J Heart Fail. 2020 Sep 23;23(1):3–12. doi: 10.1002/ejhf.1996 (PMC8287578; doi:10.1002/ejhf.1996)

**Supplemental Table 1** International Classification of Diseases 9^th^ and 10^th^ revision codes.

| **Cause of death subgroup** | **ICD-9 code** | **ICD-10 code** |
| --- | --- | --- |
| Diseases of the circulatory system | 390-459 | I00-I99 |
| Heart failure | 428 | I11,I13,I50 |
| Diseases of the respiratory system | 460-519 | J00-J99 |
| Neoplasms | 140-239 | C00-D48 |
| Other causes of death  Diseases of the digestive system | 520-579 | K00-K93 |
| Diseases of the genitourinary system | 580-629 | N00-N99 |
| Endocrine, nutritional and metabolic diseases | 240-279 | E00-E90 |
| External causes of morbidity and mortality | - | V01-Y98 |
| Mental and behavioural disorders | 290-319 | F00-F99 |
| Diseases of the nervous system | 320-359 | G00-G99 |
| Certain infectious and parasitic diseases | 001-139 | A00-B99 |
| Symptoms, signs and abnormal clinical and laboratory findings, not elsewhere classified | 780-799 | R00-R99 |
| Diseases of the musculoskeletal system and connective tissue | 710-739 | M00-M99 |
| Diseases of the skin and subcutaneous tissue | 680-709 | L00-L99 |
| Diseases of the blood and blood-forming organs | 280-289 | D50-D89 |
| Congenital malformations, deformations and chromosomal abnormalities | 740-759 | Q00-Q99 |
| Other | 360-379  380-389  800-999 | H00-H59  H60-H95  S00-T98  U00-U99 |

**Supplemental Table 2** Missing data for sociodemographic and clinical characteristics of men and women with heart failure at the time of diagnosis

| **Characteristic** | **Men** | |  | **Women** | |  |
| --- | --- | --- | --- | --- | --- | --- |
|  | **n** | **%** |  | **n** | **%** | **P-value** |
| **Overall, n (%)** | 29,234 | (100) |  | 26,725 | (100) |  |
| **Ethnic group, n (%)** |  |  |  |  |  | <0.001 |
| *White* | 23,017 | (78.7) |  | 21,126 | (79.0) |  |
| *Non-white* | 845 | (2.89) |  | 652 | (2.44) |  |
| *Mixed* | 3,636 | (12.4) |  | 2,949 | (11.0) |  |
| *Missing* | 1,736 | (5.94) |  | 1,998 | (7.48) |  |
| **Index of deprivation, n (%)** |  |  |  |  |  | <0.001 |
| *1 (least deprived)* | 5,885 | (20.1) |  | 4,969 | (18.6) |  |
| *2* | 6,876 | (23.5) |  | 6,078 | (22.7) |  |
| *3* | 6,196 | (21.2) |  | 5,751 | (21.5) |  |
| *4* | 5,972 | (20.4) |  | 5,735 | (21.5) |  |
| *5 (most deprived)* | 4,282 | (14.6) |  | 4,165 | (15.6) |  |
| *Missing* | 23 | (0.08) |  | 27 | (0.10) |  |
| **Smoking status, n (%)** |  |  |  |  |  | <0.001 |
| *Never* | 8,018 | (27.4) |  | 13,234 | (49.5) |  |
| *Former* | 4,331 | (14.8) |  | 2,763 | (10.3) |  |
| *Current* | 15,661 | (53.6) |  | 8,846 | (33.1) |  |
| *Missing* | 1,224 | (4.19) |  | 1,882 | (7.04) |  |
| **Systolic BP** |  |  |  |  |  |  |
| *Mean* ± *SD (mmHg)* | 135.4 ± 20.1 | |  | 139.9 ± 21.7 | | <0.001 |
| *Missing, n (%)* | 579 | (1.98) |  | 693 | (2.59) | <0.001 |
| **Diastolic BP** |  |  |  |  |  |  |
| *Mean* ± *SD (mmHg)* | 76.5 ± 11.6 | |  | 77.3 ± 11.6 | | <0.001 |
| *Missing, n (%)* | 579 | (1.98) |  | 693 | (2.59) | <0.001 |
| **Total cholesterol** |  |  |  |  |  |  |
| *Mean* ± *SD (mmol/L)* | 4.46 ± 4.7 | |  | 4.98 ± 1.23 | | <0.001 |
| *Missing, n (%)* | 6,613 | (22.6) |  | 8,865 | (33.2) | <0.001 |
| **Body Mass Index** |  |  |  |  |  |  |
| *Mean (SD) (kg/m2)* | 28.0 ± 5.39 | |  | 27.8 ± 6.75 | | 0.009 |
| *Missing, n (%)* | 3,479 | (11.9) |  | 4,949 | (18.5) | <0.001 |

**Abbreviations:** BMI: Body Mass Index; DBP: Diastolic Blood Pressure; HT: Hypertension; SBP: Systolic Blood Pressure;

**Supplemental Table 3** Mortality rates at one, five, 10, and 15 years after a diagnosis of heart failure by gender

| **Outcome** | **1-year MR** | **5-year MR** | **10-year MR** | **15-year MR** |
| --- | --- | --- | --- | --- |
| **All-cause mortality** |  |  |  |  |
| Men | 22.1 (21.6 to 22.6) | 44.1(43.5 to 44.7) | 52.5 (51.9 to 53.1) | 54.1 (53.5 to 54.7) |
| Women | 24.6 (24.1 to 25.1) | 46.6 (46.0 to 47.2) | 54.9 (54.3 to 55.5) | 56.4 (55.8 to 57.0) |
| **CVD mortality** |  |  |  |  |
| Men | 13.8 (13.4 to 14.2) | 25.3 (24.8 to 25.8) | 29.7 (29.1 to 30.2) | 30.4 (29.8 to 30.9) |
| Women | 15.6 (15.1 to 16.0) | 26.6 (26.1 to 27.2) | 30.5 (29.9 to 31.0) | 31.1 (30.6 to 31.7) |
| **Non CVD mortality** |  |  |  |  |
| Men | 8.23 (7.92 to 8.55) | 18.8 (18.4 to 19.3) | 22.8 (22.4 to 23.3) | 23.7 (23.2 to 24.2) |
| Women | 9.06 (8.72 to 9.41) | 20.0 (19.5 to 20.5) | 24.4 (23.9 to 24.9) | 25.3 (24.8 to 25.8) |

**Abbreviations: CV**D: Cardiovascular Disease; MR: Mortality rate

**Supplemental Table 4** CVD and non-CVD mortality rates at one, five, 10, and 15 years after a diagnosis of heart failure by gender and age category.

| **Cause of death** | **Age category** | **Gender** | **1-year MR** | **5-year MR** | **10-year MR** | **15-year MR** |
| --- | --- | --- | --- | --- | --- | --- |
| CVD | 45-54 | Men | 7.32 (5.95 to 8.68) | 12.1 (10.3 to 13.8) | 14.5 (12.6 to 16.3) | 15.0 (13.1 to 16.9) |
|  |  | Women | 3.68 (2.09 to 5.26) | 7.90 (5.63 to 10.2) | 11.0 (8.39 to 13.7) | 11.4 (8.72 to 14.1) |
|  | 55-64 | Men | 8.06 (7.19 to 8.92) | 14.9 (13.7 to 16.0) | 19.2 (17.9 to 20.4) | 20.2 (18.9 to 21.4) |
|  |  | Women | 6.56 (5.36 to 7.77) | 12.4 (10.8 to 14.1) | 15.7 (13.9 to 17.4) | 16.5 (14.7 to 18.3) |
|  | 65-74 | Men | 10.2 (9.56 to 10.9) | 20.6 (19.7 to 21.5) | 26.3 (25.3 to 27.3) | 27.6 (26.6 to 28.6) |
|  |  | Women | 9.62 (8.79 to 10.5) | 18.4 (17.3 to 19.5) | 22.9 (21.7 to 24.1) | 24.2 (23.0 to 25.4) |
|  | 75-84 | Men | 14.7 (14.0 to 15.3) | 28.1 (27.2 to 28.9) | 33.0 (32.1 to 33.8) | 33.6 (32.7 to 34.5) |
|  |  | Women | 13.3 (12.7 to 14.0) | 25.0 (24.2 to 25.8) | 29.8 (28.9 to 30.6) | 30.5 (29.7 to 31.4) |
|  | 85-94 | Men | 22.4 (21.2 to 23.5) | 36.6 (35.3 to 38.0) | 38.6 (37.3 to 40.0) | - |
|  |  | Women | 22.5 (21.6 to 23.4) | 35.5 (34.5 to 36.6) | 38.4 (37.3 to 39.4) | 38.5 (37.5 to 39.6) |
|  | 94+ | Men | 36.0 (30.2 to 41.7) | 46.1 (40.1 to 52.1) | - | - |
|  |  | Women | 33.1 (29.9 to 36.2) | 45.1 (41.8 to 48.4) | - | - |
| Non-CVD | 45-54 | Men | 2.51 (1.69 to 3.33) | 5.24 (4.07 to 6.41) | 7.10 (5.75 to 8.45) | 7.68 (6.28 to 9.07) |
|  |  | Women | 4.60 (2.83 to 6.36) | 9.93 (7.41 to 12.4) | 12.5 (9.72 to 15.3) | 14.3 (11.4 to 17.3) |
|  | 55-64 | Men | 4.09 (3.46 to 4.72) | 9.66 (8.72 to 10.6) | 12.9 (11.8 to 13.9) | 13.8 (12.7 to 14.9) |
|  |  | Women | 4.46 (3.45 to 5.46) | 12.4 (10.8 to 14.0) | 16.3 (14.5 to 18.1) | 17.8 (16.0 to 19.7) |
|  | 65-74 | Men | 5.92 (5.39 to 6.45) | 14.7 (13.9 to 15.4) | 19.6 (18.7 to 20.5) | 21.2 (20.2 to 22.1) |
|  |  | Women | 6.46 (5.77 to 7.15) | 15.2 (14.2 to 16.2) | 20.6 (19.5 to 21.8) | 22.1 (20.9 to 23.2) |
|  | 75-84 | Men | 9.48 (8.94 to 10.0) | 22.3 (21.5 to 23.1) | 26.9 (26.1 to 27.7) | 27.7 (26.9 to 28.5) |
|  |  | Women | 7.87 (7.36 to 8.39) | 19.0 (18.2 to 19.7) | 24.5 (23.6 to 25.3) | 25.5 (24.7 to 26.3) |
|  | 85-94 | Men | 13.0 (12.1 to 14.0) | 27.4 (26.2 to 28.7) | 30.2 (28.9 to 31.4) | - |
|  |  | Women | 12.3 (11.6 to 13.0) | 24.9 (23.9 to 25.8) | 28.0 (27.0 to 28.9) | 28.2 (27.2 to 29.1) |
|  | 94+ | Men | 19.9 (15.1 to 24.6) | 31.8 (26.2 to 37.5) | - | - |
|  |  | Women | 18.8 (16.2 to 21.4) | 31.9 (28.8 to 35.0) | - | - |

**Supplemental Table 5** Improvements in the survival rate over time for men and women across age groups

| **Age category** | **Gender** | **Survival** | **SR change (% per year)** | **p-value** |
| --- | --- | --- | --- | --- |
| 45-64 | Men | 1-year | 0.54 (0.32 to 0.76) | <0.001 |
|  | Women |  | 0.33 (0.01 to 0.64) | 0.043 |
|  | Men | 5-year | 1.25 (0.58 to 1.92) | 0.002 |
|  | Women |  | 0.52 (-0.20 to 1.24) | 0.138 |
|  | Men | 10-year | 2.56 (1.33 to 3.78) | 0.002 |
|  | Women |  | 1.61 (-0.27 to 3.49) | 0.081 |
| 65-74 | Men | 1-year | 0.72 (0.55 to 0.90) | <0.001 |
|  | Women |  | 0.54 (0.27 to 0.81) | <0.001 |
|  | Men | 5-year | 1.13 (0.84 to 1.43) | <0.001 |
|  | Women |  | 0.61 (0.23 to 0.99) | 0.005 |
|  | Men | 10-year | 1.88 (0.57 to 3.20) | 0.013 |
|  | Women |  | 0.83 (-0.5 to 2.15) | 0.177 |
| 75-84 | Men | 1-year | 0.73 (0.63 to 0.83) | <0.001 |
|  | Women |  | 0.39 (0.20 to 0.58) | <0.001 |
|  | Men | 5-year | 1.07 (0.85 to 1.28) | <0.001 |
|  | Women |  | 0.22 (-0.18 to 0.63) | 0.250 |
|  | Men | 10-year | 0.68 (-0.03 to 1.39) | 0.058 |
|  | Women |  | 0.39 (-0.35 to 1.13) | 0.246 |
| 85+ | Men | 1-year | 0.63 (0.24 to 1.01) | 0.003 |
|  | Women |  | 0.14 (-0.35 to 0.63) | 0.551 |
|  | Men | 5-year | 0.23 (-0.23 to 0.68) | 0.299 |
|  | Women |  | -0.05 (-0.54 to 0.44) | 0.823 |
|  | Men | 10-year | -0.16 (-0.93 to 0.62) | 0.620 |
|  | Women |  | -0.25 (-0.96 to 0.47) | 0.435 |

**Supplemental Table 6** Crude survival rates at one, five, 10, and 15 years after a diagnosis of heart failure by gender and hospitalisation at time of diagnosis

|  | **1-year SR** | **5-year SR** | **10-year SR** | **15-year SR** |
| --- | --- | --- | --- | --- |
| **Women** |  |  |  |  |
| Hospitalised | 67.0 (66.1 to 67.9) | 34.3 (33.3 to 35.4) | 15.7 (14.6 to 16.9) | 6.3 (4.76 to 8.38) |
| Non-hospitalised | 80.2 (79.6 to 80.9) | 50.9 (50.0 to 51.8) | 27.8 (26.8 to 28.8) | 13.8 (12.4 to 15.4) |
| **Men** |  |  |  |  |
| Hospitalised | 70.4 (69.6 to 71.2) | 38.9 (37.8 to 39.9) | 19.7 (18.5 to 20.9) | 9.6 (8.04 to 11.4) |
| Non-hospitalised | 82.1 (81.5 to 82.7) | 52.5 (51.7 to 53.4) | 29.8 (28.8 to 30.8) | 16.9 (15.6 to 18.3) |

**Abbreviations:** SR: Survival rate (%)

**Supplemental Figure 1** One, five and 10-year survival for men and women with heart failure by year of diagnosis


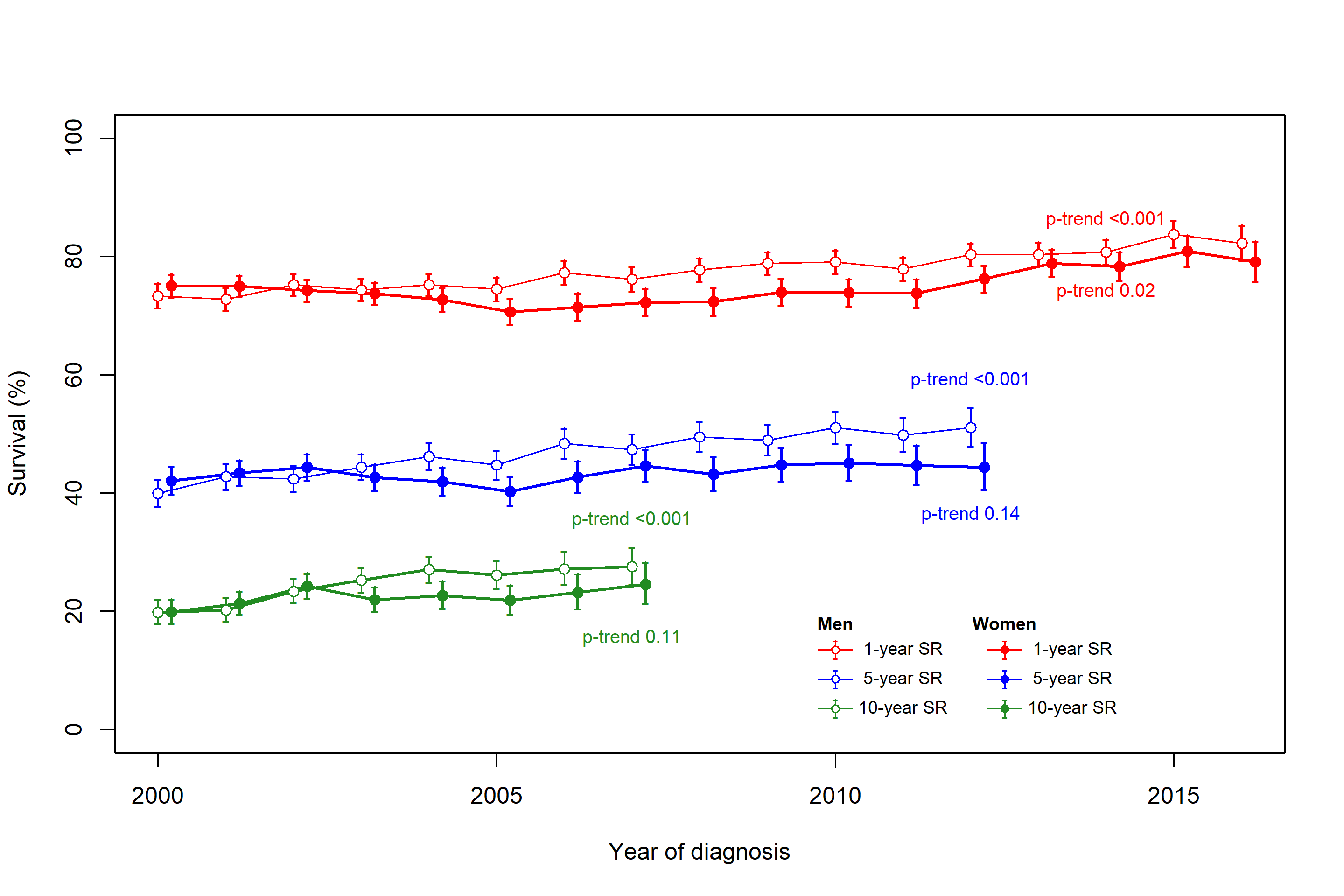


**Supplemental Figure 2** Cause-specific mortality rates over time by gender


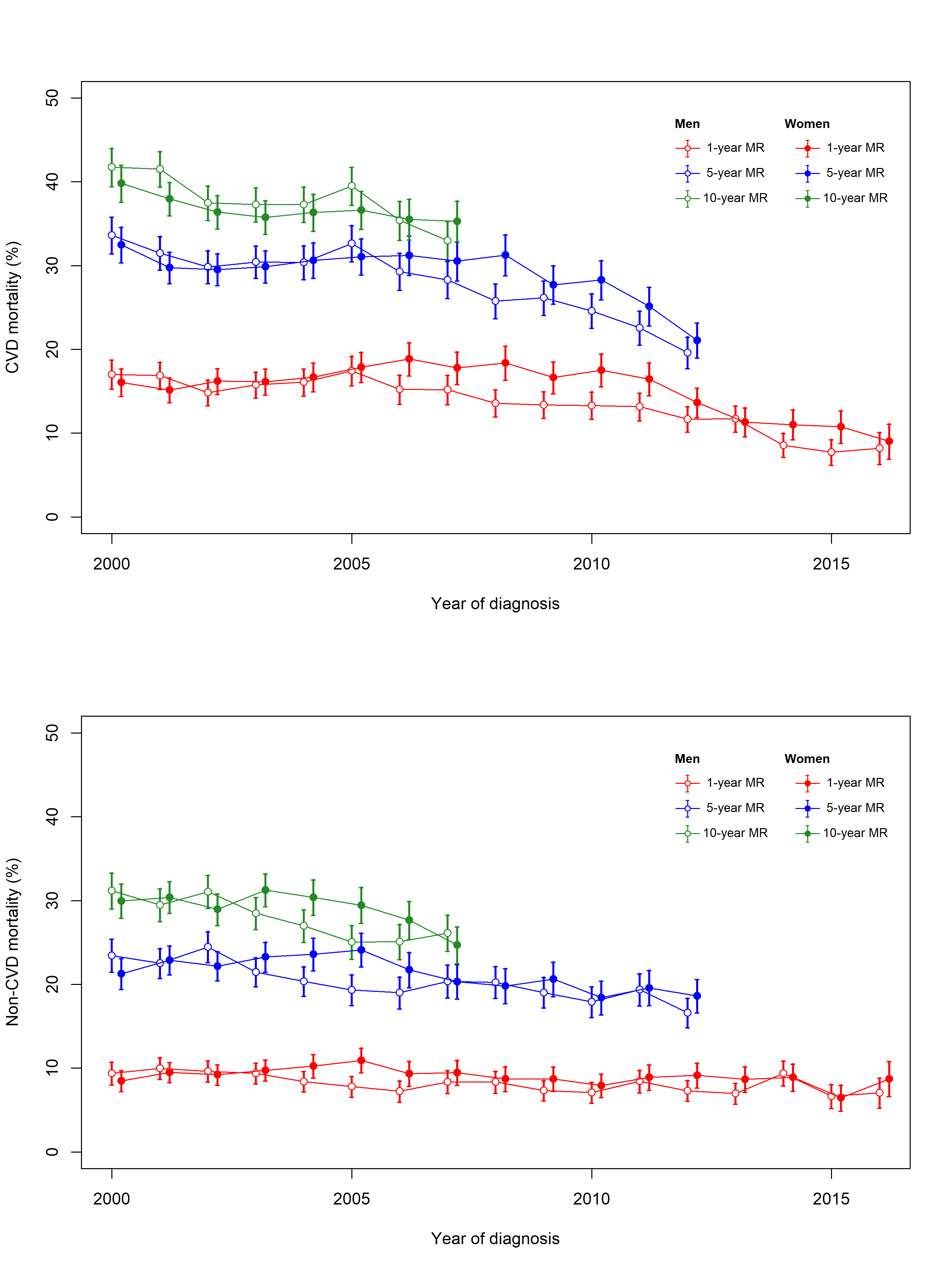

Supplement: Supplementary file 1 — Table S1. International Classification of Diseases, 9th and 10th revision codes. Table S2. Missing data for sociodemographic and clinical characteristics of men and women with heart failure at the time of diagnosis. Table S3. Mortality rates at 1, 5, 10, and 15 years after a diagnosis of heart failure by gender. Table S4. Cardiovascular disease (CVD) and non‐CVD mortality rates at 1, 5, 10, and 15 years after a diagnosis of heart failure by gender and age category. Table S5. Improvements in the survival rate over time for men and women across age groups. Table S6. Crude survival rates at 1, 5, 10, and 15 years after a diagnosis of heart failure by gender and hospitalisation at time of diagnosis. Figure S1. One, 5 and 10‐year survival for men and women with heart failure by year of diagnosis. Figure S2. Cause‐specific mortality rates over time by gender. [file EJHF-23-3-s001.docx]
